# Evaluation of diagnostic performance of SARS-CoV-2 detection kits: a comparative study

**DOI:** 10.1186/s43088-023-00360-1

**Published:** 2023-02-12

**Authors:** Basma Sherif, Hala M. Hafez, Marwa Ramadan Abdelhalim, Menna Allah Zakaria Abou Elwafa, Nancy Samir Wahba, Perihan Hamdy

**Affiliations:** 1 Clinical Pathology Department, Faculty of Medicine Ain Shams University Hospitals, Cairo, Egypt; 2 Faculty of Medicine Ain Shams University Hospitals, Cairo, Egypt

**Keywords:** COVID-19, SARS-CoV-2, Point-of-care, Rapid antigen detection, RT-PCR

## Abstract

**Background:**

Coronavirus Disease 2019 (COVID-19) pandemic has hit many countries worldwide. Rapid and accurate diagnosis is crucial to reduce disease burden. Many commercial kits have become available, but their performance needs to be assessed. This study aimed at evaluation of the diagnostic performance of real-time polymerase chain reaction (RT-PCR) and Rapid Antigen detection (RAD) kits for detecting Severe Acute Respiratory Syndrome Coronavirus 2 (SARS-CoV-2). Five hundred sixty-four Nasopharyngeal swab specimens sent to Molecular Laboratory at Ain Shams University Specialized Hospital for SARS-CoV-2 PCR testing collected from 564 subjects who attended the outpatient clinic for sample collection were randomly selected. All samples were tested for SARS-CoV-2 PCR using Viasure. Each time a recent kit was introduced, 94 samples, previously tested using Viasure, were used to determine the performance characteristics of the recent kit in comparison with Viasure, including Fast Track Diagnostics (FTD), DNA Technology, QiaPrep, Xpress SARS-CoV-2, ID NOW COVID-19 assay and Artron COVID-19 Antigen test kit.

**Results:**

Upon comparison, FTD, DNA Technology, QiaPrep, Xpress SARS-CoV-2 and ID Now showed positive percent agreement, 100%, 100%, 97.7%, 100%, 100% negative percent agreement, 86%, 100%, 98.8%, 90%, 100%, respectively. The RAD kit results, when compared with RT-PCR, showed high sensitivity at cycle threshold (Ct) < 30, low sensitivity at Ct ≥ 30, while specificity was 100%.

**Conclusion:**

Fast track, DNA Technology, QiaPrep, Xpress SARS-CoV-2 and ID Now showed good diagnostic performance. Positive RAD rule in SARS-CoV-2 infection, however negative results should be correlated with clinical condition and molecular testing.

## Background

In December 2019, multiple cases of atypical pneumonia were increasingly noticed characterized by being highly infectious with an aggressive course of illness. On 7 January 2020, the Chinese authorities announced that a novel coronavirus was identified as the implicated causative agent [10], upon which WHO reported COVID-19 outbreak a public health emergency of international concern [13]. Despite the international effort taken to prevent further transmission of infection and control its spread, the cases in different countries were increasing each day especially as a result of traveling. This increase necessitated declaration of Coronavirus Disease 2019 (COVID-19) as a pandemic on 11 Mar 2020 [6].

Highly sensitive and specific tests are crucial to identify and manage COVID-19 patients and implement control measures to limit the pandemic [1]. Viral culture and real-time reverse transcription polymerase chain reaction (RT-PCR) are the gold standards in the diagnosis of SARS-CoV-2 infection. However, both are time consuming especially viral culture, whereas RT-PCR requires special equipment and skilled laboratory personnel [14]. Many RNA gene targets can be employed in the RT-PCR, e.g., envelope (E), nucleocapsid (N), spike (S), RNA-dependent RNA polymerase (RdRp), and ORF1 genes [9]. Although being specific, alternatives to RT-PCR are essential due to the relatively long test time and the massive number of individuals needed to be tested together with shortage of resources worldwide, the availability a fast-reliable diagnostic method is a crucial demand. Rapid antigen detection (RAD) tests detect viral antigen by the immobilized coated SARS-CoV-2 antibody on the device. The test results can be interpreted without specialized instrument and available within 15 min. Hence, RAD tests can relieve the workload in hospitals and laboratories and improve the turn-around time [15]. The containment of the COVID-19 pandemic requires reliable detection of COVID-19 cases, through judicious choice of the appropriate test, taking into consideration, the relative difference in sensitivity and specificity, as well as the clinical status. [7]. Therefore, there is a critical demand for alternative detection methods, especially rapid diagnostic tests, which due to their ease of use might serve as point-of-care tests in community-based settings. Therefore, our study aimed at assessment of the diagnostic performance of multiple real-time polymerase chain reaction (RT-PCR) and Rapid Antigen detection (RAD) kits for detecting Severe Acute Respiratory Syndrome Coronavirus 2 (SARS-CoV-2) in order to cope with the intense need for COVID-19 diagnosis.

## Methods

### Sample collection

From January 2021 to October 2021, 14,000 nasopharyngeal swab specimens were collected from patients attending the outpatient clinic of Ain Shams University Hospitals. They were tested for SARS-CoV-2 PCR at the Molecular Laboratory, where nucleic acid extraction was done using Chemagic 360, followed by detection via viasure kit. Samples were collected on sterile saline 0.9% sodium chloride and preserved at 4 °C for further testing. This study included a total of randomly selected 564 specimens; 300 positive samples and 264 negative samples, 94 samples; 50 positive samples and 44 negative samples were used each time a recent kit is introduced into the laboratory to determine its performance characteristics in comparison with viasure, before testing the patient samples. Among these kits were FTD, DNA Technology, QiaPrep and Amp, ID NOW COVID-19 assay, Xpress SARS-CoV-2 Kit, Artron COVID-19 Antigen test kit. Testing was completed within a maximum of 48 h following sample collection.

### Nucleic acid detection

Viral RNA extraction and detection was performed using different commercially available methods which were compared to SARS-CoV-2 RT-PCR testing by Viasure to evaluate their performance. PCR test results were reported according to the cycle threshold (Ct) value stated by each kit. Table 1 summarizes the tested RT-PCR systems in this study.

**Table 1:** Summary of the tested RT-PCR systems in this study

Kit	Producing company	Approved by	Extraction step	Extraction performed on	Detection performed on	Detected genes	Same or different probes	Test duration (nucleic acid extraction and detection)
Viasure	PerkinElmer, USA	CE-IVD	65 min	chemagic™ 360	Bio-Rad CFX96	orf 1ab and N genes	Different probes	2 h, 45 min
FTD	Siemens healthineers, Germany	CE-IVD FDA-EUA	3 h	Versant kPCR	QuantStudio 5	orf 1ab and N genes	Same probe	5 h
DNA Technology	DNA Technology, Russia	IVD	35 min	GenePure Pro	Bio-Rad CFX96	E and N genes	Different probes	1 h, 50 min
QiaPrep & Amp	Qiagen, Germany	CE-IVD	2 min	Bio-Rad CFX96		N1 and N2 genes	Same probe	65 min
Xpert® Xpress SARS-CoV-2 Kit	Cepheid, USA	CE-IVD	Not specified	GeneXpert		N2 and E genes	Different probes	45 min
ID Now COVID-19 assay	Abbott, USA	CE-IVD FDA-EUA	Not specified	ID Now		RdRp gene		15 min

Viasure SARS-CoV-2 Detection Kit (CerTest Biotec SL, Spain): Nucleic acid extraction was done using Chemagic™ Viral DNA/RNA 300 H96 magnetic bead-based Kit utilizing chemagic™ 360 Nucleic Acid Extractor (PerkinElmer, Germany): The reverse transcription and amplification were performed on Bio-Rad CFX96 System (Bio-Rad Laboratories, Inc, USA) according to the following program: 1 cycle of reverse transcription (15 min at 45 °C), 1 cycle of initial denaturation (2 min at 95 °C), and then 45 cycles of denaturation (10 s at 95 °C), and annealing/extension (50 s at 60 °C). The kit detects orf 1ab and N genes using different probes and fluorescent dyes. Cutoff for positive results is represented by Ct value less than or equal to 40.

Fast Track Diagnostics (FTD) (Siemens healthineers, Luxembourg) where extraction is performed using Automated Versant kPCR via VERSANT Sample Preparation 1.0 Reagents Kit magnetic bead-based (Siemens healthineers, Germany): The reverse transcription and amplification were performed on Applied Biosystems™ QuantStudio 5 (Life Technologies Holdings Pte Ltd, Singapore). The kit detects orf 1ab and N genes with the same probe. Cutoff for positive results is represented by Ct value less than or equal to 38.

SARS COV-2/SARS-CoV DNA Technology (DNA Technology, Russia) after extraction using GenePure Pro (Hangzhou Bioer Technology Co. Ltd, China) via DNA TechnologyViral RNA Extraction Kit magnetic bead-based (GeneAll Biotechnology Co. Ltd, Mumbai): The reverse transcription and amplification were performed on Bio-Rad CFX96 (Bio-Rad Laboratories, Inc, USA) according to the following program: 1 cycle of reverse transcription (20 min at 35 °C), 50 cycles of denaturation (5 min at 95 °C), and annealing/extension (20 s at 64 °C). The kit detects E and N genes using different probes and fluorescent dyes. Cutoff for positive results is not specified by the kit.

QiaPrep & Amp viral RNA UM kit (Qiagen, Germany) The reverse transcription and amplification were performed on Bio-Rad CFX96 (Bio-Rad Laboratories, Inc, USA) according to the following program: 1 cycle of reverse transcription (10 min at 50 °C), 1cycle PCR initial heat activation (2 min at 95 °C), 40 cycles of denaturation (5 s at 95 °C) and annealing/ extension, as well as fluorescence acquisition (30 s at 58 °C). The kit detects N1 and N2 genes with the same probe. Cutoff for positive results is represented by Ct value less than or equal to 39.

Cepheid Xpert Xpress GeneXpert (Cepheid, USA) via Xpert® Xpress SARS-CoV-2 Kit (Cepheid, USA): based on real-time reverse transcription PCR (RT-PCR) amplification technology. The kit detects N2 and E genes using different probes and dyes, the kit considers any Ct ≥ 15 as a positive target.

ID NOW (Abbott, USA) COVID-19 assay (Abbott, USA): based on isothermal nucleic acid amplification. ID NOW gives a qualitative result based on the detection of the RNA-dependent RNA polymerase (RdRp) gene segment of SARS-CoV-2.

Artron COVID-19 Antigen test (Artron Laboratories Inc., Canada)

The swab was inserted and rotated 5–10 times in the assay diluent tube supplied by each kit, the swab was removed, then the diluent tube was closed with a filter cap and squeezed to release 3–4 drops into the sample well. The result was detected within 10–15 min.

## Results

Overall percent agreement was used to calculate the performance of the different RT-PCR and RAD kits for SARS-CoV-2 detection according to percent of positive and negative agreement with the results of RT-PCR testing via Viasure kit after nucleic acid extraction on PerkinElmer.

The Ct value which reflects viral load, in these specimens, ranged from 15 to 40, and Table 2 shows positive percent agreement (PPA) and negative percent agreement (NPA) of the tested RT-PCR kits.

**Table 2:** Results of Positive percent agreement (PPA) and Negative percent agreement (NPA) of the five PCR kits compared to Viasure

	Positive results	Positive percent agreement (%)	Negative results	Negative percent agreement (%)
FTD	50	100	38	86
DNA Technology	50	100	44	100
QiaPrep& Amp	49	98	43	97.7
Xpress SARS-CoV-2	50	100	40	90.9
ID NOW COVID-19 assay	50	100	44	100

Comparison between RAD (Artron COVID-19 Antigen test) with RT-PCR (Viasure kit) showed that the diagnostic sensitivity for Artron COVID-19 Antigen test was 100% in case of Ct value ≤ 25; 8% in case of Ct value between 25 and 40, while the diagnostic specificity was 100%.

## Discussion

This study provides a comparison for the performance of commercially available RT-PCR kits (Table 1) for the detection of SARS-CoV-2 in clinical samples. They differ regarding inclusion of extraction step, type and number of genes analyzed, sensitivity, and time needed for result release. A comprehensive study of diagnostic performance is crucial to assess accurately the kit sensitivity, where the possibility of false negatives may delay the diagnosis and leads to the premature medical discharge of infected patients (Perez and Mir, 2021).

All RT-PCR kits showed substantial agreement with Viasure, however, the interpretation of results according to cutoff value should be modified. The FTD SARS-CoV-2 assay showed 100% PPA, however, three samples that resulted negative with a high Ct using Viasure (> 40) came out to be positive using FTD with Ct thresholds (36.8, 37.7, 37.6). This result was concordant with [2], who reported substantial agreement among FTD when compared with RealStar RT-PCR kit 1.0, and reported that the discrepancies are mainly observed in specimens with relatively low amount of viral RNA. As explained by DiCarlo et al. 2021, this is attributed to detection of the ORF1ab and N genes through use of a single fluorescence probe, which permits a better sensitivity for low viral load samples (Perez and Mir, 2021).

SARS-CoV-2 RT-PCR via QiaPrep and Amp showed PPA 97.7%, NPA 98.8%, the difference in results from Viasure may be attributed to the fact that both target genes ORF 1ab and N gene were detected in Viasure using different probes, while in QiaPrep, N1 and N2 genes are detected using one probe. The use of one probe may augment the small copy number which is not detected by Viasure. However, results should be cautiously interpreted in correlation with the patient clinical status, to rule out background noise. In case of QiaPrep, additional factor may impact results, which was the duration of time between sample analysis using Viasure and QiaPrep; with a duration of maximum 4 h between both runs, the results showed high agreement, as this duration increases, discrepancy between both methods increases. This is in disagreement with [4] who reported that preservation of SARS-CoV-2 RNA in VTM is effective over 72 h. However, in their study on RAD kits, [16] demonstrated a slight decrease in the detection value with the extension of preservation time, which may explain the discrepancy observed in our study with increase in retention time. QiaPrep provides rapid extraction and detection time, where the results can be available within 90 min. In addition, it is characterized by being user-friendly and extraction equipment-free. These advantages paved the way to its use as the main RT-PCR method for sample batches at our laboratory.

DNA Technology shows high agreement with Viasure and has an advantage, as the kit detects two SARS-CoV-2 specific genes (E and N genes), the absence of one of them in the presence of the common SARS-CoV gene, highlights the probability of viral mutation. DNA Technology method detects E and N gene. E gene is prioritized for single-target testing and as a confirmatory test for COVID-19 due to the absence of abundant genomic diversity in this gene which decreases the probability of false negative results, in contrast to the RdRP and N genes which show marked diversity [3]. DNA technology provides an additional advantage of rapid extraction and detection time, where the results can be available within 120 min.

To meet the increasing demand for diagnostic tests and to offset shortages and delays in delivery of reagents, we needed a rapid and reliable molecular test for emergency cases who need hospital admission and surgical interference, in addition to emergency cases with suspicious COVID-19 for urgent diagnosis. They present the advantage of being rapid, where the results can be available within 15 and 40 min, respectively. They are used as a point-of-care testing and do not need a trained professional. Both GeneXpert and ID Now showed 100% PPA with Viasure. This is in agreement with [12] who reported GeneXpert overall PPA with cobas of 98.9%, however discordant as regard the results of ID Now, where PPA was 73.9%. Both GeneXpert and ID Now can accurately cover entire range of tested Ct values, including low-level positives. GeneXpert showed 90% NPA with Viasure in comparison with 100% for ID Now. This result is in agreement with [12] who reported a NPA between GeneXpert and cobas of 92% and for ID Now 100%. We justify this compromise in specificity of GeneXpert to be due to the high Ct cutoff level for positive results according to the manufacturer instructions. Therefore, we recommend a reconsideration for the cutoff level and to be readjusted according to the laboratory verified detection system.

Antigen tests represent a convenient way to get faster results at a lower price compared to RT-PCR assays. Our study tested the performance characteristics of the RAD (Artron COVID-19 antigen kit) tests compared to RT-PCR (using Viasure). Although the manufacturer claimed an overall sensitivity exceeding 90%, the sensitivity was variable over different viral load in tested specimens. Specimens with high viral load and Ct ≤ 25 were 100% sensitive and dropped to 8% with lower viral load. Consequently, the negative results cannot exclude SARS-CoV-2 infection confidently and thus results should be further confirmed by RT-PCR testing. Therefore, correlation with clinical findings and confirmation using molecular testing is recommended. It is worth mentioning that RAD test was applied on specimens collected on sterile saline, as those samples were primarily sent to the laboratory for PCR testing, however, RAD kits recommend collection of dry nasopharyngeal swabs, this factor needs to be furtherly investigated in another study to determine whether it contributed to the poor sensitivity or not. This poor sensitivity is concordant with [6], who reported that RAD sensitivity for detection of SARS-CoV-2 is reported to be less than both viral culture and an in-house developed RT-PCR. Also, our results are concordant with [11], where RAD test (COVID-19 Ag Respi-Strip) showed a low sensitivity, when compared to the results of RT-PCR, where, among the 106 positive samples, the COVID-19 Ag Respi-Strip detected 32 samples. For samples with Ct < 25, < 30 and < 35, COVID-19 Ag Respi-Strip has a sensitivity of 100%, 70.6% and 46.9% with an overall sensitivity of 30.2%. Regarding the high specificity of 100%, this is in agreement with previous studies reported in a review done by [5] who justified the use of antigen tests for monitoring viral clearance in hospitalized patients due to high specificity. Artron COVID-19 antigen kit detects SARS-CoV-2 nucleocapsid protein, and the use of antigen tests which target two different proteins, both outside the envelop (spike proteins) and inside the envelop (nucleocapsid proteins), is recommended to accurately detect COVID-19 variants [5].

## Conclusions

The four RT-PCR detection systems (FTD, DNA Technology, QiaPrep & Amp and Xpert Xpress) showed acceptable PPA and NPA, however, the important issue that has been highlighted through the study was that the cutoff value for positive results among different kits should be readjusted from the manufacturer instructions in relation to the verified detection system within the laboratory. This study was carried out during the SARS-CoV-2 waves and greatly helped the Molecular Microbiology laboratory within Ain Shams University hospital to cope with the great customer pressure, where our results revealed that the four RT-PCR detection systems can be used effectively for diagnosis of COVID-19 infection. The rapid PCR tests Cepheid GeneXpert and ID Now can be used in emergency cases for timely accurate diagnosis and management. Regarding RAD kit, Artron COVID-19 Antigen test can detect most of SARS-CoV-2-infected individuals with high viral load which usually represent the early stage of infection. Positive results for these antigen tests can be used as the definitive diagnosis of COVID-19 due to high specificity. However, negative results need further investigation by molecular methods. Thus, being simple, cheap and rapid test, RAD represents a good tool for diagnosis, management and follow-up of cases.

## Data Availability

The authors declare that data supporting the findings of this study are available within the article and its additional files**.**

## Abbreviations

SARS-CoV-2: Severe Acute Respiratory Syndrome Coronavirus 2
COVID-19: Coronavirus Disease 2019
VTM: Viral transport media
RT-PCR: Real-time polymerase chain reaction
RAD: Rapid Antigen detection
FTD: Fast Track Diagnostics
Ct: Cycle threshold
RdRp: RNA-dependent RNA polymerase
PPA: Positive percent agreement
NPA: Negative percent agreement

## References

[1] Corman VM, Landt O, Kaiser M, Molenkamp R, Meijer A, Chu DK, Bleicker T, Brünink S, Schneider J, Schmidt ML, Mulders DG, Haagmans BL, van der Veer B, van den Brink S, Wijsman L, Goderski G, Romette JL, Ellis J, Zambon M, Peiris M, Goossens H, Reusken C, Koopmans MP, Drosten C (2020). Detection of 2019 novel coronavirus (2019-nCoV) by real-time RT-PCR. Euro Surveill.

[2] Di Carlo D, Mazzuti L, Sciandra I, Guerrizio G, Oliveto G, Riveros Cabral RJ, Zingaropoli MA, Turriziani O (2021). Comparison of FTD SARS-CoV-2 Assay and RealStar RT-PCR kit 1.0 for the detection of SARS-CoV-2. J Virol Methods.

[3] Fukasawa LO, Sacchi CT, Gonçalves MG, Lemos APS, Almeida SCG, Caterino-de-Araujo A (2021). Comparative performances of seven quantitative Reverse-Transcription Polymerase Chain Reaction assays (RT-qPCR) for detecting SARS-CoV-2 infection in samples from individuals suspected of COVID-19 in São Paulo, Brazil. J Clin Virol Plus.

[4] Garnett L, Bello A, Tran KN, Audet J, Leung A, Schiffman Z, Griffin BD, Tailor N, Kobasa D, Strong JE (2020). Comparison analysis of different swabs and transport mediums suitable for SARS-CoV-2 testing following shortages. J Virol Methods.

[5] Kyosei Y, Yamura S, Namba M, Yoshimura T, Watabe S, Ito E (2021). Antigen tests for COVID-19. Biophys Physicobiol.

[6] Mak GC, Cheng PK, Lau SS, Wong KK, Lau CS, Lam ET, Chan RC, Tsang DN (2020). Evaluation of rapid antigen test for detection of SARS-CoV-2 virus. J Clin Virol.

[7] Mohammadi M, Meskini M, do Nascimento Pinto AL (2020). 2019 Novel coronavirus (COVID-19) overview. Z Gesundh Wiss.

[8] Pérez-López B, Mir M (2021). Commercialized diagnostic technologies to combat SARS-CoV2: advantages and disadvantages. Talanta.

[9] Puck B, van der Bas V, van den Sharon B, LisaW JJ, Annemarie B, Richard M, Chantal R, Adam M (2020). Comparison of seven commercial RT-PCR diagnostic kits for COVID-19. J Clin Virol.

[10] Read J, Bridgen J, Cummings D, Ho A, Jewell C (2020). Novel coronavirus 2019-nCoV: early estimation of epidemiological parameters and epidemic predictions. medRxiv.

[11] Scohy A, Anantharajah A, Bodéus M, Kabamba-Mukadi B, Verroken A, Rodriguez-Villalobos H (2020). Low performance of rapid antigen detection test as frontline testing for COVID-19 diagnosis. J Clin Virol.

[12] Smithgall MC, Scherberkova I, Whittier S, Green DA (2020). Comparison of Cepheid Xpert Xpress and Abbott ID Now to Roche cobas for the rapid detection of SARS-CoV-2. J Clin Virol.

[13] WHO (2020) COVID-19 public health emergency of international concern (PHEIC) global research and innovation forum, 12 February 2020. https://www.who.int/publications/m/item/covid-19-public-health-emergency-of-international-concern-(pheic)-global-research-and-innovation-forum

[14] WHO (2020) Laboratory testing for coronavirus disease 2019 (COVID-19) in suspected human cases: interim guidance, 2 March. https://apps.who.int/iris/handle/10665/331329

[15] WHO (2020) Laboratory testing strategy recommendations for covid-19: interim guidance, 21 March. https://apps.who.int/iris/handle/10665/331509

[16] Zhou H, Wang C, Rao J, Chen L, Ma T, Liu D, Ren L, Xu S (2021). The impact of sample processing on the rapid antigen detection test for SARS-CoV-2: Virus inactivation, VTM selection, and sample preservation. Biosaf Health.

